# Optimizing Taq Polymerase Concentration for Improved Signal-to-Noise in the Broad Range Detection of Low Abundance Bacteria

**DOI:** 10.1371/journal.pone.0007010

**Published:** 2009-09-15

**Authors:** Rudolph Spangler, Noel L. Goddard, David S. Thaler

**Affiliations:** 1 Sackler Laboratory of Molecular Genetics and Informatics, The Rockefeller University, New York, New York, United States of America; 2 Department of Physics & Astronomy, Hunter College, City University of New York, New York, New York, United States of America; 3 Howard Hughes Medical Institute, Albert Einstein College of Medicine, Bronx, New York, United States of America; Baylor College of Medicine, United States of America

## Abstract

**Background:**

PCR in principle can detect a single target molecule in a reaction mixture. Contaminating bacterial DNA in reagents creates a practical limit on the use of PCR to detect dilute bacterial DNA in environmental or public health samples. The most pernicious source of contamination is microbial DNA in DNA polymerase preparations. Importantly, all commercial Taq polymerase preparations inevitably contain contaminating microbial DNA. Removal of DNA from an enzyme preparation is problematical.

**Methodology/Principal Findings:**

This report demonstrates that the background of contaminating DNA detected by quantitative PCR with broad host range primers can be decreased greater than 10-fold through the simple expedient of Taq enzyme dilution, without altering detection of target microbes in samples. The general method is: For any thermostable polymerase used for high-sensitivity detection, do a dilution series of the polymerase crossed with a dilution series of DNA or bacteria that work well with the test primers. For further work use the concentration of polymerase that gave the least signal in its negative control (H_2_O) while also not changing the threshold cycle for dilutions of spiked DNA or bacteria compared to higher concentrations of Taq polymerase.

**Conclusions/Significance:**

It is clear from the studies shown in this report that a straightforward procedure of optimizing the Taq polymerase concentration achieved “treatment-free” attenuation of interference by contaminating bacterial DNA in Taq polymerase preparations. This procedure should facilitate detection and quantification with broad host range primers of a small number of bona fide bacteria (as few as one) in a sample.

## Introduction

PCR in principle can detect a single target molecule in a reaction mixture. Contaminating bacterial DNA in reagents creates a practical limit on the use of PCR to detect dilute bacterial DNA in environmental or public health samples. The most pernicious source of contamination is microbial DNA in DNA polymerase preparations.

Trace bacterial DNA in Taq polymerase is hard to eliminate because of the enzyme's bacterial source as well as reagents and equipment used in its purification. This is a problem for the detection of bacterial DNA when two specific conditions apply: 1) Target DNA is present in very small quantities; and 2) Bacterial species are unknown, possibly mixed, and the assay cannot be designed to exclude specific species. The ideal assay for unbiased monitoring and discovery applications should be both sensitive and general; i.e., one wants the ability to broadly detect any bacterial species and retain the ability to identify it. 16S PCR primers as close to universal as possible seem ideal for the purpose. In cases where bacterial DNA is detected, it can be further characterized by sequencing using the same primers.

A number of studies have indicated the presence of microbial DNA in commercial preparations of heat-stable DNA polymerases [1]–[6]. The DNA sequencing of cloned DNA from amplicons by Hughes et al., in 1994 [5], confirmed the earlier restriction enzyme analysis of Rand and Houck, in 1990 [1], which showed that the source of bacterial DNA contamination was not from *E. coli* or *T. aquaticus*. It is generally thought that some step(s) in the purification, or reagents added to the enzyme, are the sources of this bacterial contamination. Such contaminants from manufacturing equipment are even found in sterile products produced for clinical applications, such as mouth swabs [7].

Estimates of contamination in commercially available lots of recombinant Taq polymerase range from 10–1000 genome equivalents of bacterial DNA per Unit of enzyme [1], [4]–[6]. A problem not rigorously addressed in reports on quantification of contaminating DNA is that the efficiency of PCR assays on DNA of unknown sequence cannot be determined. Specifically, it is difficult to assign a quantity for a template for which there is no standard curve.

Removal of DNA from an enzyme preparation is problematical. Sensitive detection of PCR product with Real-time qPCR calls into question some of the earlier reports, using end-point detection on agarose gels, of “complete” removal of contaminating DNA. In some cases, it is not clear whether reduction of contaminating signal was from removal of contaminating DNA or from inhibition of polymerase activity from the treatment applied. With qPCR technology it is possible to include controls with added DNA to sensitively test the effects of treatments on amplification, as well as the effects of treatments on removing background DNA [6].

No universally applicable method for removing DNA from Taq polymerase preparations has been established [6]. Treatment with ultraviolet light below 320 nm (UVB or UVC) is effective at making DNA resistant to amplification [8], but ultraviolet light that inactivates DNA also reduces the efficiency of Taq polymerase [4], [6], [9]–[11]. Application of ultrafiltration with Millipore filters (YM100) was reported to reduce Taq polymerase sensitivity in one case [12], but not in another [13]. Our experiments with “Hot-start” Taq polymerases containing antibody inactivators indicated that the Taq polymerase associated with the antibody did not pass through the YM100 filter (data not shown).

UVA-activated psoralen treatment to reduce DNA in Taq polymerase preparations has been applied with variable results [5], [6], [14]. The number of variables required to be optimized for this method are daunting: preincubation time with the Taq preparation must allow psoralens to localize with DNA; the psoralen concentration must be sufficient to inactivate the DNA after exposure to UVA, but not so high that it will affect the subsequent PCR reaction; time of exposure to UVA and intensity of UVA must be established; the optimum for these variables could change from lot-to-lot of Taq polymerase.

Restriction endonuclease digestion [6], [12], [15], [16], and digestion with DNase I [6], [12], [17]–[22], have been used to reduce DNA background in Taq polymerase preparations with variable results. Removal of DNA from mastermix containing DNA polymerase by DNase treatment introduces a number of complications, particularly with respect to elimination of DNase. While DNase I can be inactivated by elevated temperature, it might be capable of renaturing when the temperature is reduced. Results with DNase I cleanup of DNA from mastermix are highly variable. For example, heating for 95°C for 50 min to inactivate the DNase reduces the effectiveness of the polymerase [22]. Reducing the temperature for inactivation to 80°C with addition of a reducing agent (dithiothreitol) was reported to reduce contamination, but this method still requires addition of a DNase I buffer high in magnesium [23].

One (typical) Unit of *Taq* DNA polymerase incorporates 10 nmoles of deoxyribonucleotide into acid-precipitable material in 30 min at 74°C. The specific activity of most commercial preparations of Taq is ∼80,000 Units/mg of protein [24]. For a 94 kDalton Taq polymerase this would be ∼8x10^10^ Taq molecules per Unit. From the values cited above it is clear that a PCR reaction with the typical one Unit of Taq polymerase starts with a vast excess of polymerase molecules compared to DNA template.

A reasonable hypothesis is that contaminating DNA will decrease linearly with dilution of the enzyme preparation, while the amplification ability on a small number of targets will remain constant, even with less Taq polymerase, until some point at which the geometrically increasing target DNA approaches the Taq polymerase abundance. This was found to be the case in the present studies, providing a simple method to decrease background noise by dilution of Taq polymerase, and increasing the reliability of qPCR assays for low-level bacterial DNA.

## Results

**Figure 1:**
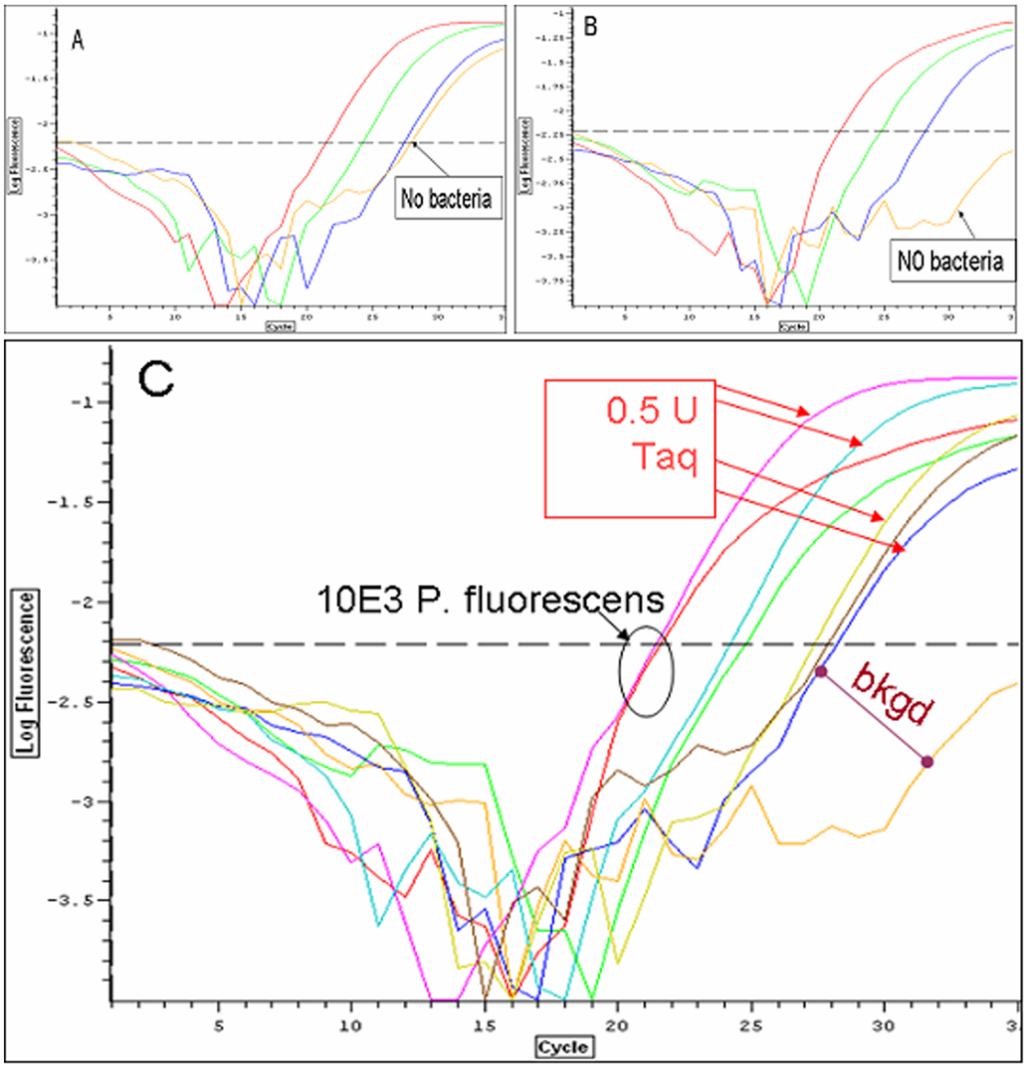
Detection of *Pseudomonas fluorescens* at high and low Taq polymerase concentrations. Bacterial detection with 0.5 Units (A) or 0.05 Units (B) Qiagen Taq DNA polymerase with 16S350B assay on samples containing 10^3^, 10^2^, 10^1^ and zero *Pseudomonas fluorescens* bacteria. A composite of A and B is shown in C.

Fig. 1A shows a series of amplifications carried out using 0.5 Units of Taq polymerase. The short dilution series of *Pseudomonas fluorescens* includes an important negative control containing no bacteria in the reaction. Fig. 1B is the same dilution series carried out with one-tenth the amount of Taq polymerase. As seen in the composite Fig. 1C, diluting the Taq polymerase 10-fold did not alter the amplification of *Pseudomonas fluorescens* bacteria spiked into the reaction, as measured by the cycle at which reactions reached threshold. Importantly, in the absence of added target bacteria (No bacteria), the 10-fold dilution of the Taq polymerase led to a large reduction in amplification of “background,” indicating that the target DNA creating this background signal from the 16S rDNA assays was present in the Taq polymerase preparation. Similar results were seen for most other polymerases tested.

**Figure 2:**
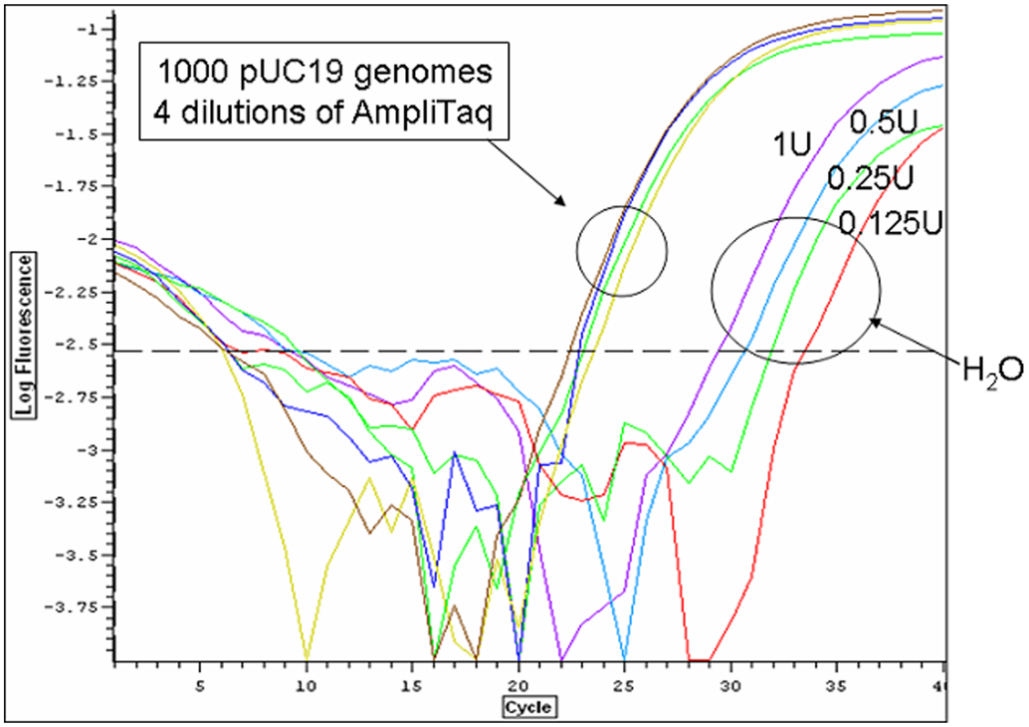
Detection of the beta-lactamase gene in commercial Taq polymerase. Four dilutions of Amplitaq DNA polymerase were tested with primers for the beta-lactamase gene in the presence of 10^3^ pUC19 plasmids (labeled 1000 pUC19 genomes) or H_2_O.

As shown in Fig. 2, Taq-associated DNA could be detected corresponding to the beta-lactamase gene of pUC19. Of the 8 polymerases tested with both 16S and beta-lactamase assays, only 1 (Roche FastStart) did not generate a reliable signal with the beta-lactamase primers.

**Figure 3:**
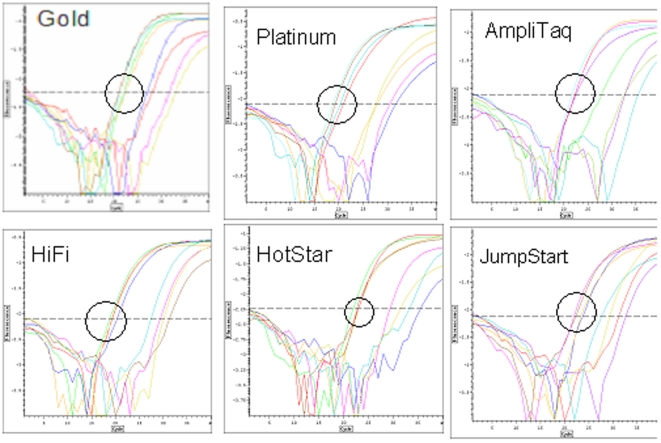
Detection of bacterial DNA in six Taq polymerases. Four dilutions of six DNA polymerase were tested with primers for 16S rDNA in the presence of 100 pg (∼10̂5 16S rDNA) E. coli genomic DNA (circled) or H_2_O.

Fig. 3 shows the results of serial 2-fold dilutions of 6 different Taq polymerases. There was no significant difference for the 4 dilutions in detection of a fixed amount (100 pg) of E. coli DNA. However, the signal in the absence of spiked E. coli DNA was reduced predictably with dilution for all Taq polymerases.

**Figure 4:**
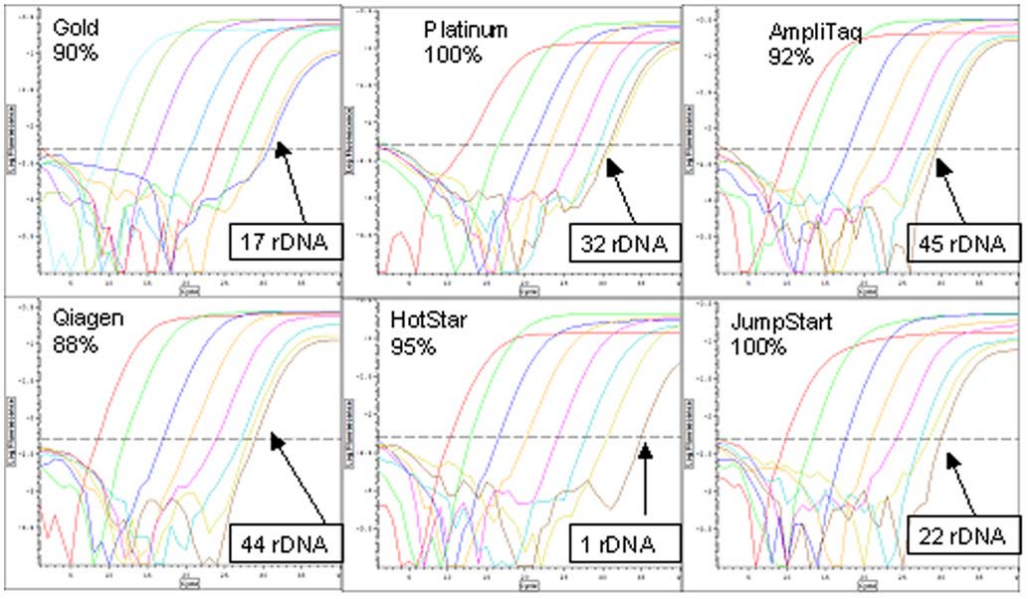
Approximating the copy number of 16S rDNA in commercial Taq polymerases. Six DNA polymerases were used with primers for 16S rDNA on 7 serial 10-fold dilutions of E. coli genomic DNA (10 ng to 10 fg) with no added DNA in the 8^th^ sample. The least squares fit equation for each dilution series was used to assign a value to the signal from the 8^th^ sample, which contains Taq-associated DNA only. The efficiency of the reaction was determined from the slope of the linear fit plotting the base10 log of the DNA concentration vs. the threshold cycle. A slope of −3.322 indicates an average doubling rate of “2,” which is approximately 100% efficiency (2̂3.322∼10). The rDNA values assigned are for “E. coli equivalents.”

Fig. 4 shows the results of serial 10-fold dilutions of E. coli DNA assayed with 16S rDNA primers using different Taq polymerases. The efficiency of the amplification was determined from the slope of the least squares fit for each polymerase, and the amount of (E. coli equivalent) rDNA in the zero DNA sample was predicted from the fit equation for each set of serial dilutions. Because the DNA in the zero DNA well is not the same as E. coli DNA (except in the case of the contamination in AmpliTaq), the E. coli equivalent values possibly underestimate the amount of contaminating DNA.

**Figure 5:**
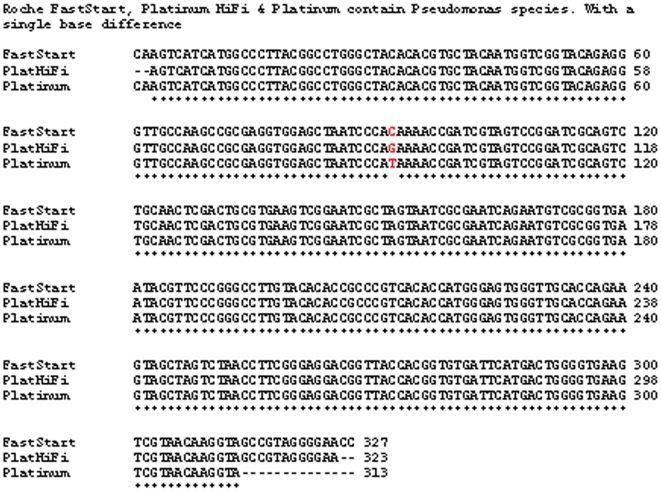
Sequences of contaminating bacterial DNA in three Taq polymerases. Sequence alignment of three contaminants from different commercial Taq polymerases showing the presence of different strains of the same Pseudomonas species. Roche FastStart, Platinum HiFi & Platinum Taq polymerases contain similar strains of a Pseudomonas species with a single base difference in the region covered by the 16S350 PCR assay.

Fig. 5 shows the sequence alignments of DNA from three Taq polymerases with similar contaminants. All three are Pseudomonas species by BLAST analysis. Other Taq polymerases had distinct contaminants (not shown). HotStar contained DNA that aligned with *Serratia marcescens* and other “phytobacteria.” Amplitaq contained DNA that aligned with *Escherichia coli*, Salmonella and Shigella. Qiagen Taq DNA polymerase contained a mixture of contaminants that did not give reliable sequencing data from reaction to reaction.

**Figure 6:**
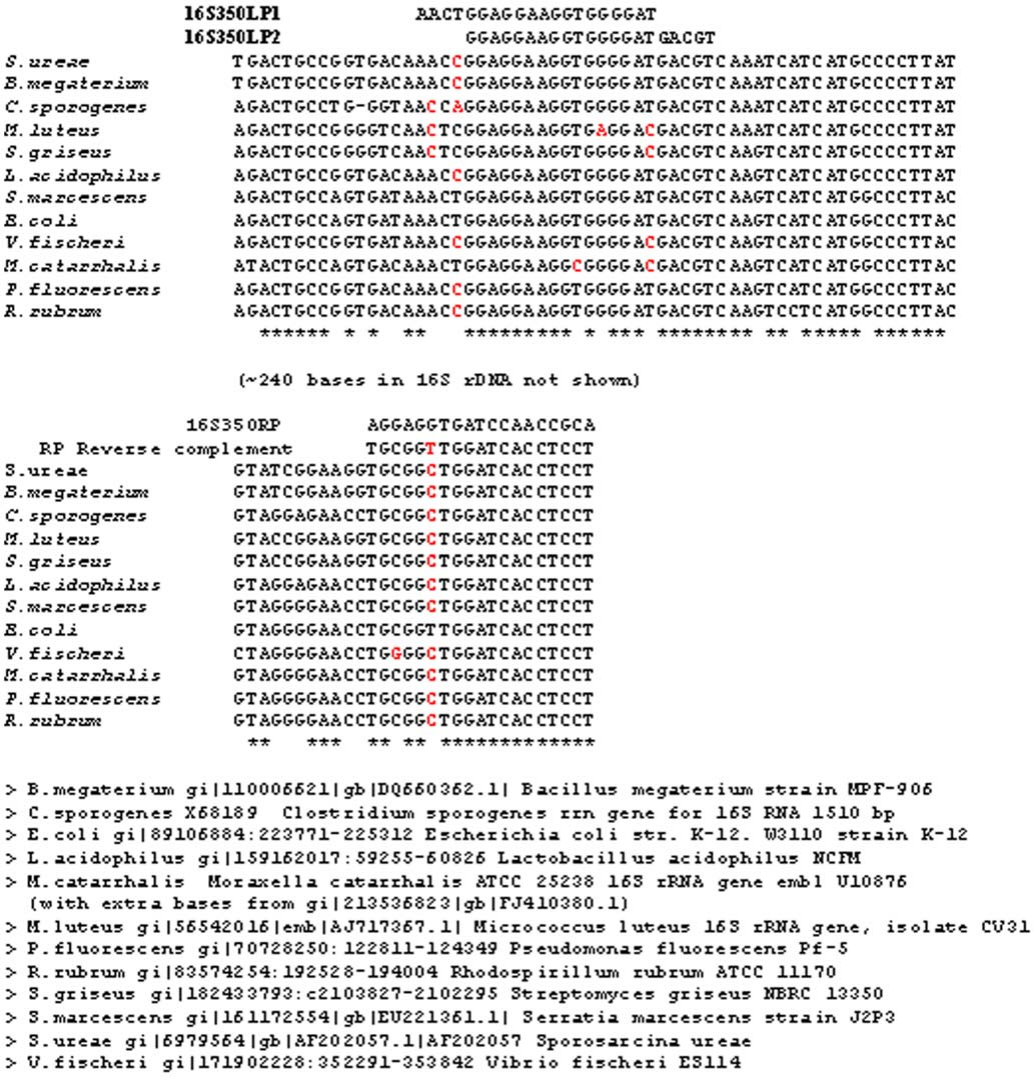
Alignment of 16S primers on the genomes of 12 bacterial sequences. Four bacteria, *M.luteus, S.griseus, V.fischeri* and *M.catarrhalis* were detected at least at 100-fold greater levels with LP2 than with LP1, presumably due to the lack of basepairing of the 3′ base of LP1. The key beneath the figure provides the GenBank records for the sequences shown.

Fig. 6 shows the alignment of the broad host range primers for bacterial 16S rDNA used in this study. These primers were tested against the commercially available bacteria described in Fig. 6. The two 16S rDNA forward primers cover a similar conserved region, offset by four or five bases (shown in bold): 16S350LP1, **AACT** GGAGGAAGGTGGGGAT; 16S350LP2, GGAGGAAGGTGGGGAT**GACGT** . A single reverse primer was used with each of the forward primers in separate reactions. The reverse primer (S16S350RP, AGGAGGTGATCCAACCGCA) binds approximately 370 bases downstream of the forward primers on most bacterial genomes. Because some species have a mismatch at the 3′ end of 16S350LP1 (Figure 6), Taq polymerase samples were assayed with both 16S350LP1 and 16S350LP2.

## Discussion

In an important paper that remains relevant, Corless et al address sources of contamination in 16S assays and assess several methods for purifying Taq preparations [6]. In the end, they appear to acknowledge a noble failure in their Abstract: “Without the development of ultrapure *Taq* DNA polymerase, ultrapure reagents, and plasticware guaranteed to be free of DNA, the implementation of a PCR for detection of eubacterial 16S rRNA by sensitive technologies, such as the TaqMan system, will continue to be problematical.”

It is clear from the studies shown in this report that the simple expedient of diluting the Taq polymerase preparation is able to accomplish in a straightforward manner what more elaborate procedures by numerous workers (see introduction for references) failed to accomplish: The reliable “treatment-free” attenuation of interference by contaminating bacterial DNA in Taq polymerase preparations. This procedure should facilitate detection and quantification with broad host range primers of a small number of bona fide bacteria (as few as one) in a sample.

The response of qPCR to dilution of Taq has several notable features:

1. The background level of DNA contamination in the water controls decreases with dilution.

2. The threshold cycle (Ct) for the detection of added bacterial DNA is similar for low and high Taq concentrations. The variation with Taq polymerase dilution on spiked DNA or spiked bacteria (1,000 or 10,000 targets) was not significant up to 27- or 32-fold dilution of Taq polymerase. This conclusion is based on a comparison of the variation between duplicate measures at the same dilution with the variation among dilution-to-dilution measures (data not shown). The variability for duplicate measures at each dilution was as great as the variability among the different dilutions (in the presence of sufficient target DNA). This is consistent with the prediction that, even with dilution, there are in the early cycles of the PCR, a vast excess of polymerase molecules with respect to the number of targets.

3. Although the cycle at which the reactions reach the Ct is unaffected by dilution, the behavior of the amplification profiles after the reactions pass the threshold cycle can be affected by enzyme dilution at reasonably low levels of target DNA. The amplification curves with diluted enzyme cross the threshold at the same point and with a similar slope as those made with enzyme at higher concentration. As geometric amplification cannot go on indefinitely, the slopes of amplification curves in reactions with less Taq polymerase begin to decrease before the slopes in reactions with more Taq. After passing the threshold cycle, the slopes of diluted enzyme amplification curves decrease a number of cycles earlier than those with a higher concentration of enzyme. Consequently, for reactions with a fairly low level of initial spiked target, the absolute fluorescence in reactions with more Taq reaches a higher plateau than reactions with less Taq.

Two points can be made, one practical and one theoretical. From a practical point of view the method of diluting the polymerase only works with real-time qPCR. If the sample is assayed with conventional end-point PCR and the results visualized on gels, diluted polymerase generates a lower signal for the same amount of added target (not shown).

The theoretical point is a quantitative speculation about what happens near and after the threshold cycle. Calculations estimate that a single unit of Taq contains ca 8×10^10^ molecules of polymerase. Consider Fig. 1 in which approximately 10 bacteria reach the threshold cycle at Ct ∼27 whether or not the enzyme is diluted 10-fold. The efficiency of the Qiagen Taq in that reaction buffer is about 88%, and after 27 cycles each starting molecule would be increased by (1.88)^27^ or ca. 2.5×10^7^. Starting with the predicted 10 *P*. *fluorescens* bacteria in that well, and assuming there are 6 rDNA molecules per genome, the number of templates at Ct 27 is reasonably 2.5×10^7^×10 genomes ×6 rDNA/genome  = 1.5×10^9^ rDNA templates. At this point, the template concentration is about 4% of the Taq polymerase concentration, assuming no loss of polymerase during the 27 cycles. In 0.5 unit of polymerase there are about six cycles left before the ratio of estimated template and estimated polymerase reach unity. In the reaction with 10-fold diluted enzyme, the estimated number of enzyme molecules is closer to 8×10^9^. The number of target amplicons at 27 cycles (Ct) is the same at ∼1.8×10^9^ rDNA, however it takes only 3 cycles after Ct in order to approach theoretical molar equality to the number of polymerase molecules. This is consistent with the notion that Taq enzyme is still in excess over template in the diluted Taq reaction after 27 cycles (1.8×10^9^ templates and 8×10^9^ Taq molecules).

The numbers are crude estimates but they are consistent enough with the data to indicate that nothing mysterious is occurring. The system behaves the way it does because when there is a large excess of enzyme over target there is no difference in amplification between the two concentrations of Taq polymerase. However, when enzyme and target start to approach molar equality, the increase in product at each cycle necessarily shifts from geometric to arithmetic amplification.

The dilution approach might not have worked for any number of reasons, including the possibility that DNA in the Taq polymerase might not have been the major contributor to background amplification. Importantly, contributions to background from all possible sources in these studies besides the Taq polymerase preparation turned out to be non-significant. With all the other possible sources of contamination eliminated (including water, buffer salts, dye, oligonucleotides), optimization of polymerase concentration provides a simple way to obtain more reliable results.

A similar strategy might be applied to other programs that rely on amplification of target DNA, such as whole genome sequencing from single cells from the environment [25]. Optimization of polymerase concentration to reduce contaminating DNA from multiple displacement amplification (MDA) polymerase used for single cell sequencing might simplify bioinformatics assembly of microbial genomes [26], [27]. For detection of possible microbial DNA contaminants in the Phi29 polymerase used for MDA for example, it might be helpful to heat inactivate the phage polymerase and test small aliquots for the presence of microbial 16S rDNA. If 16S rDNA is detected, the Phi29 polymerase could be diluted prior to amplification of target DNA.

In summary, the signal to noise ratio of qPCR for the detection of eubacterial DNA can be improved significantly by diluting thermostable polymerases. The general method is: For any thermostable polymerase used for high-sensitivity detection, make a dilution series of the polymerase crossed with a dilution series of DNA or bacteria that work well with the test primers. For further work use the concentration of polymerase that gave the lowest signal in its negative control (H_2_O) without changing the threshold cycle for spiked DNA or bacteria compared to higher concentrations of Taq polymerase.

## Materials and Methods

### Taq polymerases

The following DNA polymerases from commercial vendors designed for qPCR were used in the experiments reported here: Amplitaq Gold (ABI, CA; Roche lot # J02913); Platinum Taq (Invitrogen, CA; cat # 10966–026; lot 1169610); Platinum HiFi Taq (Invitrogen, CA; cat# 11304–011; lot# 1267490); HotStar Taq (Qiagen, CA; Mat # 1007837; lot # 124125007); JumpStart Taq (Sigma, MO; cat # D-6558; lot # 71K9029). Two polymerases not designed specifically for qPCR (no anti-Taq antibody) were also tested: Amplitaq DNA polymerase (ABI, CA; Roche lot # C00622); Qiagen Taq DNA polymerase (Qiagen, CA; Cat # 201205, lot # 127132149. Additional development studies were carried out with a 2x FastStart Universal SYBR Green I Master Mix (Roche, IN; cat # 04913922001).

### PCR reaction buffer and cycling conditions

A common master mix was prepared to test all Taq polymerases. Final concentrations of components in the reaction were: 20 mM TRIS (pH 8.2); 50 mM KCl; 3 mM MgCl_2_; 375 uM each dNTP; 1% DMSO; 5% glycerin; and 20,000-fold diluted SYBR Green I (Molecular Probes, Invitrogen, Carlsbad CA). Similar results were obtained using the ABI Gold Taq PCR Core Components (ABI, cat# 4304886). PCR reactions were carried out in a final volume of 10 ul on Biorad's Chromo4 Four Color Real Time PCR Detector with Gradient DNA Engine Thermocycler. PCR reactions were initiated with a 94°C heating step for 10 min. Cycling was then carried out with melting at 94°C, 10 sec; annealing at 60°C, 10 sec; extension at 72°C, 20 sec. After 35 or 40 cycles, dissociation analysis was carried out from 60°C to 94°C, with ramping at 0.5°C per minute.

### Dilutions of Taq polymerases

Stock solutions of Taq polymerase at 5 U/ul were diluted in 2- or 3-fold increments in 2 ng/ul linear acrylamide. 2.5 ul of each dilution were added to 2.5 ul of 4x mastermix with 2.5 ul oligonucleotide primers (300 nM final each primer) and 2.5 ul of sample or H_2_O.

### Samples

Standard curves were generated by 10-fold or 5-fold serial dilution of E. coli DNA (cat. # D4889, Sigma-Aldrich, St. Louis MO) for 16S rDNA quantification. pUC19 DNA was similarly diluted for beta-lactamase quantification (Invitrogen, Carlsbad CA). Dilutions were carried out in the presence of 2 ng/ul linear acrylamide as carrier. Dilutions were tested starting from as high as 10 ng E. coli DNA (approximately 1.2×10^7^ copies of 16S rDNA). Similar serial dilutions were also tested for the 2686 bp pUC19 DNA starting with as much as 1 pg plasmid (approximately 3.44×10^5^ copies of the pUC19 plasmid containing the beta-lactamase gene). The bacteria listed in Fig. 6 were purchased as a set of 12 from Carolina Biological Supply Company, Burlington, NC (Cat# 154706). Bacterial samples diluted in water were spiked directly into PCR reaction wells and heated to 94°C prior to commencing thermal cycling.

### qPCR Assays

All oligonucleotides were synthesized by Integrated DNA Technologies, Inc. (IDTDNA.com; Coralville, Iowa). The desalted oligonucleotides were suspended in 5 mM Tris (pH 8) with 0.1 mM EDTA. The two 16S rDNA forward primers cover a similar conserved region, offset by four or five bases (shown in bold): 16S350LP1, **AACT** GGAGGAAGGTGGGGAT; 16S350LP2, GGAGGAAGGTGGGGAT**GACGT** . A single reverse primer was used with each of the forward primers in separate reactions. This primer (S16S350RP, AGGAGGTGATCCAACCGCA) binds approximately 370 bases downstream of the forward primers on most bacterial genomes. These primers were tested against the commercially available bacteria described in Figure 6. The alignments of the two left primers and one right primer on the 16S rDNA of these bacteria are shown in Figure 6. The 16S350 primers amplify a region of approximately 370 bases in the 16S rDNA of most bacteria. Because some species have a mismatch at the 3′ end of 16S350LP1 (Figure 6), samples were assayed with both 16S350LP1 and 16S350LP2. Primers for beta lactamase were B_Lac_LP, AATAAACCAGCCAGCCGGAA, and B_LAC_RP, CGGAGGACCGAAGGAGCTAA. The beta lactamase (BLac) primers amplify a region of 267 bases in the BLac gene of the pUC19 plasmid (GenBank accession # L09137).

### Sequencing of contaminants

All sequencing was carried out by Gene-Wiz Inc. (South Plainfield, NJ). PCR reactions were purified on QIAquick PCR Purification columns (Qiagen Cat. # 28104). Cleanup column loading buffer contains ethanol, which removes the SYBR Green I dye from the DNA. The amplicons binding to the column were eluted in water and the OD determined prior to adding appropriate amounts to tubes containing either left or right primer. Sequencing was carried out from both sides of the amplicon and the overlapping results were merged (omitting the primer sequences, which do not necessarily reflect the precise sequence on the contaminating target).

## References

[1] Rand KH, Houck H (1990). Taq polymerase contains bacterial DNA of unknown origin.. Mol Cell Probes.

[2] Boettger EC (1990). Frequent contamination of Taq polymerase with DNA.. Clin Chem.

[3] Schmidt TM, Pace B, Pace NR (1991). Detection of DNA contamination in Taq polymerase.. Biotechniques.

[4] Meier A, Persing DH, Finken M, Bottger EC (1993). Elimination of contaminating DNA within polymerase chain reaction reagents: implications for a general approach to detection of uncultured pathogens.. J Clin Microbiol.

[5] Hughes MS, Beck LA, Skuce RA (1994). Identification and elimination of DNA sequences in Taq DNA polymerase.. J Clin Microbiol.

[6] Corless CE, Guiver M, Borrow R, Edwards-Jones V, Kaczmarski EB (2000). Contamination and sensitivity issues with a real-time universal 16S rRNA PCR.. J Clin Microbiol.

[7] Iversen BG, Eriksen HM, Bo G, Hagestad K, Jacobsen T (2007). Pseudomonas aeruginosa contamination of mouth swabs during production causing a major outbreak.. Ann Clin Microbiol Antimicrob.

[8] Spangler R, Goddard NL, Spangler DN, Thaler DS (2009). Tests of the Single-hit DNA Damage Model.. J Mol Biol.

[9] Ou CY, Moore JL, Schochetman G (1991). Use of UV irradiation to reduce false positivity in polymerase chain reaction.. Biotechniques.

[10] Goldenberger D, Altwegg M (1995). Eubacterial PCR - contaminating DNA in primer preparations and its elimination by UV-light.. Journal of Microbiological Methods.

[11] Harris KA, Hartley JC (2003). Development of broad-range 16S rDNA PCR for use in the routine diagnostic clinical microbiology service.. J Med Microbiol.

[12] Mohammadi T, Reesink HW, Vandenbroucke-Grauls CM, Savelkoul PH (2003). Optimization of real-time PCR assay for rapid and sensitive detection of eubacterial 16S ribosomal DNA in platelet concentrates.. J Clin Microbiol.

[13] Yang S, Lin S, Kelen GD, Quinn TC, Dick JD (2002). Quantitative multiprobe PCR assay for simultaneous detection and identification to species level of bacterial pathogens.. J Clin Microbiol.

[14] Jinno Y, Yoshiura K, Niikawa N (1990). Use of psoralen as extinguisher of contaminated DNA in PCR.. Nucleic Acid Research.

[15] Sharma JK, Gopalkrishna V, Das BC (1992). A simple method for elimination of unspecific amplifications in polymerase chain reaction.. Nucleic Acids Res.

[16] Carroll NM, Adamson P, Okhravi N (1999). Elimination of bacterial DNA from Taq DNA polymerases by restriction endonuclease digestion.. Journal of Clinical Microbiology.

[17] Furrer B, Candrian U, Wieland P, Luthy J (1990). Improving PCR efficiency.. Nature.

[18] Rochelle PA, Weightman AJ, Fry JC (1992). DNase I treatment of Taq DNA polymerase for complete PCR decontamination.. Biotechniques.

[19] Hilali F, Saulnier P, Chachaty E, Andremont A (1997). Decontamination of polymerase chain reaction reagents for detection of low concentrations of 16S rRNA genes.. Molecular Biotechnology.

[20] Lyons SR, Griffen AL, Leys EJ (2000). Quantitative real-time PCR for Porphyromonas gingivalis and total bacteria.. J Clin Microbiol.

[21] Klaschik S, Lehmann LE, Raadts A, Hoeft A, Stuber F (2002). Comparison of different decontamination methods for reagents to detect low concentrations of bacterial 16S DNA by real-time-PCR.. Molecular Biotechnology.

[22] Heininger A, Binder M, Ellinger A, Botzenhart K, Unertl K (2003). DNase pretreatment of master mix reagents improves the validity of universal 16S rRNA gene PCR results.. Journal of Clinical Microbiology.

[23] Silkie SS, Tolcher MP, Nelson KL (2008). Reagent decontamination to eliminate false-positives in Escherichia coli qPCR.. J Microbiol Methods.

[24] Sambrook J, Russel DW (2001). Molecular cloning. A laboratory manual..

[25] Ishoey T, Woyke T, Stepanauskas R, Novotny M, Lasken RS (2008). Genomic sequencing of single microbial cells from environmental samples.. Curr Opin Microbiol.

[26] Stepanauskas R, Sieracki ME (2007). Matching phylogeny and metabolism in the uncultured marine bacteria, one cell at a time.. Proc Natl Acad Sci U S A.

[27] Woyke T, Xie G, Copeland A, Gonzalez JM, Han C (2009). Assembling the marine metagenome, one cell at a time.. PLoS One.

